# Long-term risk of adverse events in patients discharged alive after hospitalization for hypertensive crisis

**DOI:** 10.1097/HJH.0000000000004113

**Published:** 2025-08-06

**Authors:** Tommaso Bucci, Steven H.M. Lam, Antonios A. Argyris, D. Gareth Beevers, Eduard Shantsila, Alena Shantsila, Gregory Y.H. Lip

**Affiliations:** a Liverpool Centre for Cardiovascular Science at University of Liverpool, Liverpool John Moores University and Liverpool and Heart and Chest Hospital, Liverpool, UK; b Department of Clinical Internal, Anesthesiologic and Cardiovascular Sciences, Sapienza University of Rome, Rome, Italy; c Cardiovascular Prevention and Research Unit, Clinic/Laboratory of Pathophysiology, Laiko Hospital, Medical School, National and Kapodistrian University of Athens, Greece; d University of Birmingham, Department of Medicine, City Hospital, Birmingham; e Department of Primary Care and Mental Health; f Department of Cardiovascular and Metabolic Medicine, University of Liverpool, Liverpool, UK; g Department of Clinical Medicine, Aalborg University, Aalborg, Denmark

**Keywords:** emergency, hypertension crisis, target organ damage, urgency

## Abstract

**Objective:**

To evaluate the long-term clinical course of patients presenting with hypertensive crisis discharged alive from hospital.

**Methods:**

Retrospective study utilizing TriNetX. Based on the ICD-10-CM codes recorded between 2000 and 2022, patients with hypertensive crisis were subdivided into hypertensive urgencies (HU) and hypertensive emergencies (HE). In those with HE, the type of target organ damage was reported, i.e. central nervous system (ischemic or haemorrhagic stroke and hypertensive encephalopathy), cardiovascular (myocardial infarction (MI), heart failure (HF), aorta dissection (AD)), or renal (acute kidney failure). Primary outcomes were the one-year risks of all-cause death, and major cardiovascular events (MACE: MI, stroke, cardiac arrest, AD, and HF). Secondary outcomes were the risks for each type of MACE and the incident risk of Atrial Fibrillation (AF). Cox regression analysis after propensity score matching (PSM) 1 : 1 was used to produce hazard ratios (HRs) and 95% Confidence Intervals (95%CIs).

**Results:**

Overall, we identified 27 721 patients with HE (age 62.4 ± 15.7, 46.3% females) and 23 478 patients with HU (age 63.4 ± 17.3, 55.8% females). After PSM, patients with HE showed a higher risk of all-cause death [hazard ratio (HR), 1.33, 95% confidence internal (CI) 1.24–1.44] and MACE (HR 4.00, 95% CI 3.79–4.22), vs. those with HU. Of the secondary outcomes, patients with HE had increased risks of MI, stroke, cardiac arrest, AD, acute HF, AD and incident AF. All the different types of organ involvement were associated with similar long-term risks of adverse events. During follow-up, 4% of patients with HU progressed to HE. Young age, female sex, Black or Asian ethnicity, smoking, secondary hypertension, diabetes and chronic kidney disease were identified as the main risk factors.

**Conclusion:**

Patients with HE have a high long-term risk of all-cause death, MACE and incident AF. Preventing the onset of target organ damage in patients with hypertensive crisis is crucial to mitigate their long-term risk of adverse events.

## INTRODUCTION

Hypertensive crisis is defined as a severe and rapid increase of the blood pressure (BP) ≥180/110 mmHg that often requires immediate but careful intervention to lower BP [1]. This clinical condition often needs the access to the emergency department and can be further classified into hypertensive urgency or emergency based on the presence of acute target organ damage [2]. Hypertensive urgencies are characterized by a rapid and severe BP elevation without organ injuries, whereas hypertension emergencies involve acute target organ damage, including the central nervous system (hypertensive encephalopathy and ischemic or haemorrhagic stroke), the cardiovascular system [myocardial infarction (MI), heart failure (HF), and aorta dissection or rupture], or the kidneys (acute renal failure) [3].

Although there has been significant overall improvement in survival after hypertensive crises over the past fifty years [4], both hypertensive urgency and emergency are still associated with an increased short-term risks of adverse events [5]. Relatively few studies on small populations have investigated the long-term risk of adverse events in patients who have survived hypertensive crises [6–8], and no data are available about the impact of the number and type of target organ damage and their associated risks of adverse clinical outcomes. Our aim was to assess the one-year risk of adverse events in patients presenting with hypertensive crises, using a large global federated healthcare dataset.

## METHODS

### Study design

This retrospective observational study was conducted within TriNetX, a federated health research network that accesses electronic medical records from various participating healthcare organizations, such as academic medical centres, specialty physician practices, and community hospitals. This network covers a population of approximately 250 million individuals. Data available within TriNetX include demographics, diagnoses coded using the International Classification of Diseases, Ninth Revision and Tenth Revision, Clinical Modification (ICD-10-CM), and medications coded using the Veteran Affairs Codes. For further details, visit the following link: https://trinetx.com/company-overview/.

TriNetX operates as a health research network in compliance with the Health Insurance Portability and Accountability Act (HIPAA) and relevant United States (US) federal laws safeguarding healthcare data privacy and security. This includes adherence to the HIPAA Privacy Rule's de-identification standard for healthcare data. Access requires submission of requests to the platform and execution of a data sharing agreement. Since TriNetX functions as a federated research network, studies utilizing its resources do not necessitate ethical approval, as they do not involve receipt of personally identifiable patient information. Additional details regarding data extraction can be found in the supplementary materials.

### Cohort

The searches on the TriNetX online research platform were performed on the 24th of February 2025 for individuals aged ≥18 years who were discharged from the hospital after a hypertensive crisis. Based on the ICD-10-CM codes recorded within one month before the discharge, patients were subdivided in two groups: hypertensive urgencies (I16.0), and hypertensive emergencies (I16.1). In those with hypertensive emergencies, the type of target organ damage was reported, i.e. central nervous system [CNS: ischemic stroke (I63), hypertensive encephalopathy (I67.4), intracerebral haemorrhages (I60 and I61)], cardiovascular [CV: myocardial infarction (MI (I21)], acute heart failure (HF (I50.21, I50.23, I50.31, I50.33, I50.41, I50.43), aortic dissection or rupture (AD (I71.1, I71.3, I71.5, I71.8)), or renal (acute kidney failure (N17)). More information about the ICD-10-codes utilized can be found in Supplementary Table 1.

The study period was comprised between the 1 January 2000 and 31 December 2022. At the time of the search, 85 participating healthcare organizations, primarily located in the US, had data available for patients who met the study inclusion criteria. The baseline index event date was the discharge from hospital, any other diagnoses or treatment reported before this date were the individual's baseline characteristics.

### Outcomes

The primary outcomes were the one-year risk of: all-cause death, and major Adverse Cardiovascular Events (MACE), i.e. ischemic stroke, MI, acute HF, cardiac arrest, aortic dissection or rupture. The secondary outcomes were the one-year risk of each component of the MACE outcome. The adverse events of interest were identified via ICD-10-CM codes (Supplementary Table 2).

### Statistical analysis

All statistical analyses were performed on the TriNetX online research platform. Propensity score matching (PSM) 1 : 1 with greedy nearest neighbour algorithm was used to create balanced cohorts. Absolute standardized mean differences (ASD) were used to show the distribution of demographic and clinical data among the groups and was calculated as the difference in the means or proportions of a particular variable divided by the pooled estimate of standard deviation for that variable. Any baseline characteristic with an ASD < 0.1 was considered well matched. We included the following variables in the PSM: age, sex, ethnicity, essential hypertension, secondary hypertension, hypertensive CKD, hypertensive chronic heart disease, dyslipidaemia, diabetes, chronic ischemic heart diseases, obesity, atrial fibrillation (AF), occlusion or stenosis of carotid arteries, occlusion or stenosis of cerebral arteries, chronic heart failure, cardiomyopathies, pulmonary embolism, and cardiovascular medications (including β-blockers, antiarrhythmics, diuretics, antilipemic agents, antianginals, calcium channel blockers, angiotensin-converting enzyme inhibitors, angiotensin II receptor blockers, and antiplatelets). After PSM, univariable Cox proportional hazard models were used to calculate hazard ratios (HRs) and 95% confidence intervals (95% CIs) for the risk of adverse events in patients with hypertensive emergencies compared to those with hypertensive urgencies. Kaplan-Meier survival curves and the Log-Rank test were also generated to illustrate differences in event-free survival distributions between patients with hypertensive emergency and hypertensive urgency.

Exploratory analyses were performed, to assess the one-year risk of secondary outcomes, excluding those patients who experienced the event of interest before the index event. In addition to the outcomes used in the main analysis, this exploratory investigation also estimates the risk of incident AF and the need for dialysis.

Moreover, we assessed the risk of primary outcomes based on the prevalent type of target organ damage during the hypertensive emergency and in those with all the three clinical manifestations (i.e. CNS, CV and renal).

Additionally, we investigated the reproducibility of the results obtained from the main analysis—comparing patients with hypertensive emergency with those with hypertensive urgency—by performing the comparison separately within each subgroup: sex (men vs women), age (<75 or ≥75 years), presence or absence of chronic kidney disease (CKD, ICD code N18), AF (ICD code I48) and the most represented ethnic groups (White, Black or African, and Asian). Interaction analysis was conducted by comparing HRs between subgroups using a Z-test on the difference of log-transformed HRs, with standard errors derived from the 95% CIs of the HRs.

Lastly, among patients with hypertensive urgency, the incidence of hypertensive emergencies during the one-year follow-up after hospital discharge was recorded. An exploratory multivariable Cox regression analysis was then performed exclusively in this group to identify independent predictors of progression to hypertensive emergency. The model was adjusted for female sex, age ≥65 years, ethnicity (White, Black or African American, and Asian), alcohol abuse, smoking, primary and secondary hypertension, obesity, dyslipidaemia, diabetes, and CKD.

All tests were two-tailed and *P*-values of <0.05 were taken to indicate statistical significance. TriNetX does not impute or estimate clinical values to fill gaps in a patient's record. All analyses were performed in the TriNetX platform which uses R's survival package v3.2–3.

## RESULTS

Overall, we included 27 721 patients with hypertensive emergencies (mean age 62.4 ± 15.7 years, 46.3% females), and 23 478 patients with hypertensive urgencies (mean age 63.5 ± 17.2 years, 55.7% females). Patients with hypertensive emergencies were more likely men, Black or African American and had a higher prevalence of chronic hypertensive complications and cardiovascular risk factors (Table 1). After PSM, no significant differences were found between the two groups (Table 1).

**TABLE 1:** Baseline characteristics of patients with hypertensive emergency and urgency before and after the propensity score matching

	Before PSM			After PSM		
	Hypertensive emergencies (*n* = 27 721)	Hypertensive urgencies (*n* = 23 478)	ASD	Hypertensive emergencies (*n* = 17 191)	Hypertensive urgencies (*n* = 17 191)	ASD
Age	62.3 ± 15.8	63.4 ± 17.3	0.064	63.0 ± 15.9	62.9 ± 17.1	0.004
Female sex	12 816 (46.2)	13 091 (55.8)	0.191	8940 (52.0)	8932 (52.0)	0.001
White	12 595 (45.4)	13 416 (57.1)	0.236	8985 (52.3)	8965 (52.1)	0.003
Black or African American	9388 (33.9)	6053 (25.8)	0.177	4943 (28.8)	4989 (29.0)	0.001
Asian	1871 (6.7)	1150 (4.9)	0.079	1001 (5.8)	961 (5.6)	0.010
Essential hypertension	24 126 (87.0)	20 311 (86.5)	0.015	15 061 (87.6)	15 048 (87.5)	<0.001
Secondary hypertension	2103 (7.6)	1463 (6.2)	0.053	1210 (7.0)	1192 (6.9)	0.004
Hypertensive CKD	8203 (29.6)	4837 (20.6)	0.208	4425 (25.7)	4272 (24.9)	0.020
Hypertensive heart disease	7743 (27.9)	3217 (13.7)	0.356	3086 (18.0)	3031 (17.6)	0.008
Chronic ischemic heart disease	11 120 (40.1)	7054 (30.0)	0.210	5986 (34.8)	5950 (34.6)	0.004
Occlusion and stenosis of carotid artery	3630 (13.1)	1931 (8.2)	0.158	1789 (10.4)	1753 (10.2)	0.007
Occlusion and stenosis of cerebral arteries	888 (3.2)	234 (1.0)	0.154	260 (1.5)	232 (1.3)	0.014
Pulmonary embolism	804 (2.9)	856 (3.6)	0.042	541 (3.3)	556 (3.2)	0.005
Atrial fibrillation and flutter	4916 (17.7)	3488 (14.9)	0.078	2831 (16.5)	2774 (16.1)	0.009
Diabetes mellitus	12 414 (44.8)	8972 (38.2)	0.134	7133 (41.5)	7155 (41.6)	0.003
Obesity	9268 (33.4)	7246 (30.9)	0.055	5423 (31.5)	5449 (31.7)	0.003
Dyslipidaemia	17 213 (62.1)	13 288 (56.6)	0.112	10 305 (59.9)	10 288 (59.8)	0.002
Chronic systolic heart failure	2266 (8.2)	828 (3.5)	0.199	860 (5.0)	782 (4.5)	0.021
Chronic diastolic heart failure	3071 (11.1)	1920 (8.2)	0.199	1699 (9.9)	1624 (9.4)	0.015
Beta Blockers	25 494 (92.0)	18 789 (80.0)	0.349	15 166 (88.2)	15 246 (88.7)	0.015
Calcium channel blockers	23 246 (83.9)	16 756 (71.4)	0.303	13 698 (79.7)	13 782 (80.2)	0.007
Diuretics	19 705 (71.1)	13 276 (56.5)	0.306	10 856 (63.1)	10 887 (63.3)	0.004
Statins	20 118 (72.6)	13 071 (55.7)	0.358	11 173 (65.0)	11 172 (65.0)	<0.001
Antiarrhythmics	17 768 (64.1)	151 215 (64.4)	0.007	11 199 (65.1)	11 014 (64.1)	0.023
Antianginals	14 381 (51.9)	8515 (36.3)	0.318	7361 (42.8)	7328 (42.6)	0.004
ACE inhibitors	15 929 (57.5)	11 927 (50.8)	0.134	9376 (54.5)	9335 (54.3)	0.005
Angiotensin II receptor antagonist	10 070 (36.3)	8052 (34.3)	0.042	6200 (36.1)	6140 (35.7)	0.007
Anticoagulants	24 193 (87.3)	19 387 (82.6)	0.132	14 601 (84.9)	14 525 (84.5)	0.012
Antiplatelet	20 084 (72.5)	13 456 (57.3)	0.321	11 211 (65.2)	11 234 (65.3)	0.003

ASD, absolute standardized mean difference; CKD, chronic kidney disease; PSM, propensity score matching.

**FIGURE 1:**
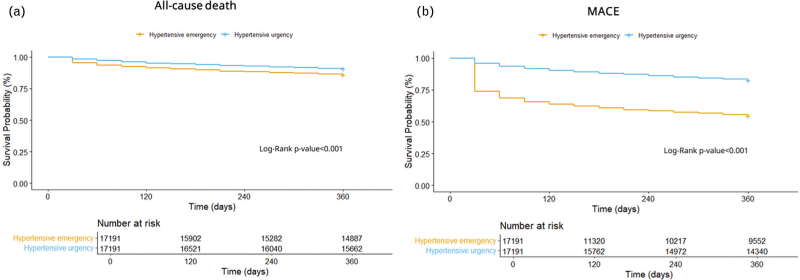
Kaplan–Meier curves for the risk of all-cause death (panel a) and major adverse cardiovascular events (panel b), in patients with hypertensive emergency compared to those with hypertensive urgency. MACE, major adverse cardiovascular events.

The total number of events recorded in each group after one-year follow-up is reported in Table 2. After PSM, patients with hypertensive emergencies were associated with a higher one-year risk of all-cause death (HR, 1.33, 95% CI 1.24–1.44, Fig. 1, panel a) and MACE (HR 4.00, 95% CI 3.79–4.22, Fig. 1, panel b) compared to those with hypertensive urgencies. There was also increased risk of secondary outcomes. Hypertensive emergencies were associated with higher one-year risks of MI (HR 3.93, 95% CI 3.54–4.36), stroke (HR 4.57, 95% CI 4.20–4.98), cardiac arrest (HR 1.36, 95% CI 1.12–1.65), aortic dissection or rupture (HR 5.83, 95% CI 4.20–8.10), and acute HF (HR 2.76, 95% CI 2.52–3.01), when compared to patients with hypertensive urgencies.

### First cardiovascular events

On the exploratory analysis performed to investigate the risk of first CV events, the high risks of primary and secondary outcomes in patients with hypertensive urgencies was consistent with the main analysis. For aortic dissection or rupture this was not statistically significant due to small numbers and wide 95%CIs although the point estimate was suggestive of an increased risk (Table 2).

**TABLE 2:** Risk of primary and secondary outcomes in patients with hypertensive emergency

	Overall analysis after PSM			Excluding patients with the outcome of interest before the time window		
	Hypertensive emergencies (*n* = 17 191) n (%)	Hypertensive urgencies (*n* = 17 191) *n* (%)	HR (95%CI)	Hypertensive emergencies number of patients / number of events (%)	Hypertensive urgencies number of patients / number of events (%)	HR (95%CI)
All-cause death	1529 (8.9)	1197 (7.0)	1.33 (1.24–1.44)	–	–	–
MACE	5575 (32.4)	1746 (10.2)	4.00 (3.79–4.22)	5230 / 558 (10.7)	13 876 / 850 (6.1)	1.91 (1.71–2.12)
Myocardial infarction	1634 (9.5)	450 (2.6)	3.93 (3.54–4.36)	12 439 / 285 (2.3)	16 014 / 248 (1.5)	1.55 (1.31–1.84)
Stroke	2708 (15.8)	665 (3.9)	4.57 (4.20–4.98)	11 599 / 493 (4.3)	15 724 / 364 (2.3)	1.92 (1.68–2.20)
Cardiac arrest	262 (1.0)	211 (1.2)	1.36 (1.12–1.65)	16 983 / 206 (1.2)	17 047 / 181 (1.1)	1.19 (0.71–1.45)
Aortic dissection or rupture	237 (1.4)	42 (0.2)	5.83 (4.20–8.10)	16 807 / 25 (0.2)	17 128 / 21 (0.1)	1.26 (0.71–2.26)
Acute heart failure	1760 (10.2)	692 (4.0)	2.76 (2.52–3.01)	13 133 / 387 (3.0)	15 938 / 430 (2.7)	1.14 (1.00–1.31)
Atrial fibrillation^a^	–	–	–	14 359 / 365 (2.5)	14 389 / 321 (2.2)	1.18 (1.01–1.37)
Dialysis^a^	–	–	–	16 152 / 248 (1.6)	15 438 / 185 (1.2)	1.38 (1.14–1.67)

CI, confidence interval; HR, hazard ratio; MACE, major adverse cardiovascular events; PSM, propensity score matching.

a Analysis performed only in patients without these conditions before the index event.

After excluding patients with preexisting AF, the one-year risk of incident AF in 14 359 patients with hypertensive emergencies and 14 389 well matched patients with hypertensive urgencies was 2.5% and 2.2%, respectively (*P* = 0.032). Time-adjusted risk estimation confirmed a higher risk of incident AF in patients with hypertensive emergencies compared to those with hypertensive urgencies (HR 1.18, 95% CI 1.01–1.37).

### Sensitivity analysis

**FIGURE 2:**
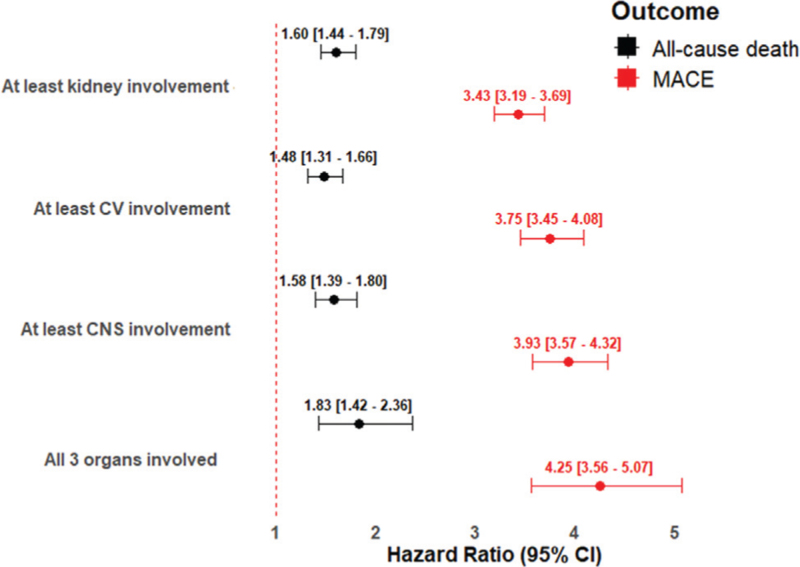
Risk of primary outcomes according to the type of target organ damage in patients with hypertensive emergencies compared to those with hypertensive urgencies. CI, confidence interval; CNS, central nervous system; CV, cardiovascular; MACE, major adverse cardiovascular events. The risk of primary outcomes is reported as the hazard ratio (outside the brackets) and its 95% confidence interval (inside the brackets) after propensity score matching.

On the sensitivity analysis performed to evaluate the risk of primary outcomes in patients with hypertensive emergencies based on the type of the prevalent acute organ damage, we found that all the three possible target organ damages were associated with a similar increased risk of adverse events after the discharge with no substantial difference compared to those with all the three acute manifestations (Fig. 2 and Supplementary Table 3).

### Subgroup analysis

**FIGURE 3:**
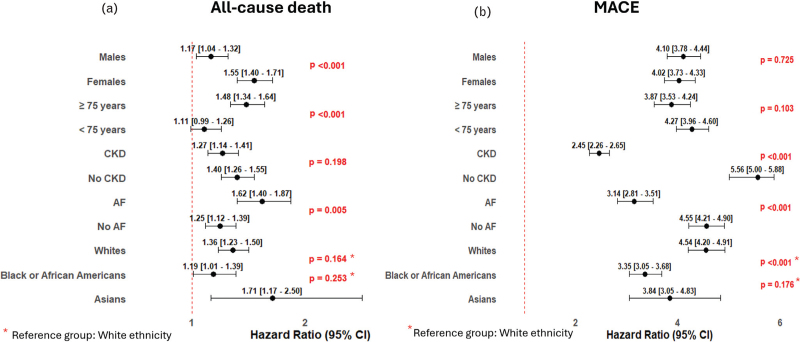
Subgroup analyses for the risk of all-cause death (panel a) and major adverse cardiovascular events (panel b). CV, cardiovascular; CNS, central nervous system; MACE, major adverse cardiovascular events. The risk of primary outcomes is reported as the hazard ratio (outside the brackets) and its 95% confidence interval (inside the brackets) after propensity score matching. The *P* value (in red) refers to the interaction analysis.

The risks of all-cause death and MACE in patients with hypertensive emergencies was irrespective of sex, ethnicity (White, Black African or Asian), age < or ≥ 75 years, and presence or absence of CKD or AF (Fig. 2, panels a and b, Supplementary Table 4). The risk of all-cause death was higher in patients females (HR 1.55, 95% CI 1.40–1.71) compared to males (HR 1.17, 95% CI 1.04–1.32), in those with hypertensive emergencies aged ≥75 years (HR 1.48, 95% CI 1.34–1.64) compared to those aged <75 years (HR 1.11, 95% CI 0.99–1.26), and in those with AF (HF 1.62, 95% CI 1.40–1.87) compared to those without AF (HR 1.25, 95% CI 1.12–1.39). The risk of MACE was higher in patients with hypertensive emergencies without CKD (HR 5.56, 95% CI 5.00–5.88), or AF (HR 4.55, 95% CI 4.20–4.91) or White (HR 4.54, 95% CI 4.20–4.91) compared to those with CKD (HR 2.45, 95% CI 2.26–2.65), with AF (HR 3.14, 95% CI 2.81–3.51), or Black or African American (HR 3.35, 95% CI 3.05–3.68), respectively (Fig. 3).

### Exploratory analysis of the risk of Progression to hypertensive emergency following hypertensive urgency

During the one-year postdischarge risk period, in the overall population of patients with hypertensive urgency before PSM, 960 patients (4.1%) progressed to hypertensive emergency.

On exploratory multivariable Cox regression analysis (Supplementary Figure 1), factors independently associated with an increased risk of progression to hypertensive emergency included female sex (HR 1.24, 95% CI 1.13–1.37), Black or African American ethnicity (HR 1.54, 95% CI 1.32–1.80), Asian ethnicity (HR 1.32, 95% CI 1.03–1.70), smoking (HR 1.63, 95% CI 1.47–1.80), secondary hypertension (HR 1.46, 95% CI 1.28–1.66), diabetes (HR 1.23, 95% CI 1.11–1.36), and CKD (HR 3.68, 95% CI 3.33–4.07). Conversely, factors associated with a significantly reduced risk of progression were advanced age (HR 0.53, 95% CI 0.47–0.59) and obesity (HR 0.78, 95% CI 0.71–0.86).

## DISCUSSION

In this study, our principal findings are as follows: patients with hypertensive emergencies exhibited a distinct clinical phenotype, characterized by a high prevalence of male sex, Black or African American ethnicity, preexisting hypertension and other cardiovascular risk factors; patients with hypertensive emergencies were associated with a higher risk of death, MACE and incident AF and dialysis compared to those with hypertensive urgencies; the risk of primary outcomes in patients with hypertensive emergencies was similar regardless of the type of target organ damage and was independent of age, sex, ethnicity and preexisting CKD or AF; during the year after discharge from hospital 4.1% of patients with hypertensive urgency progress to hypertensive emergency, with most important risk factors represented by young age, female sex, Black and Asian ethnicity, smoking, secondary hypertension, diabetes and CKD.

It has been already reported that hypertension crises tend to be more frequent in males and certain ethnicities, such as Blacks or African Americans [9,10]. A retrospective study using data from the Nationwide Inpatient Sample (NIS) showed that in the US, during the period between 2002 and 2014, the overall incidence rate for hypertensive crises was around 2 new cases per 100 000 individuals per year [10]. Moreover, the same study showed that the odds of experiencing hypertensive crisis requiring hospitalization increased annually, for both men and women, with a higher increase in men compared to women [odds ratio (OR), 1.083 per year; 95% CI, 1.078– 1.09 vs. 1.077 per year, 95% CI, 1.070– 1.079] [10]. This was also confirmed by a retrospective study from Italy involving more than 330 000 patients admitted to the emergency department [11]. This study reported that, compared to women of similar age, men had higher likelihood of experiencing hypertensive emergencies rather than urgencies (OR 1.34, 95% CI 1.06–1.70), independent of presenting symptoms, creatinine, smoking habit and known hypertension [11]. Additionally, a retrospective study from the Netherlands, which enrolled patients admitted to the hospital for hypertensive crises over a period of 12 years (1993–2005), showed that Black African or Afro-Caribbean ethnicity was associated with a fourfold increased incidence of hypertensive crises (7.3 new cases per 100 000 individuals per year) compared to Caucasians [9].

Regarding the high cardiovascular risk factors burden found in patients with hypertensive emergencies, it is well known that hypertensive crises often occur in hypertensive patients, particularly those who are noncompliant with therapeutic regimens or are prescribed ineffective treatments [12]. In this context, hypertensive crises could result from the interactions among the different factors. The presence of uncontrolled hypertension may predispose individuals to developing further cardiovascular risk factors such as CKD, coronary artery disease, and diabetes which increase the arterial stiffness and further worsen BP control. The coexistence of several comorbidities, associated with polypharmacy and frailty, could complicate compliance with complex pharmacological treatments. Moreover, the high prevalence of certain ethnicities in studies conducted in Western countries suggest that, in addition to race-related pathophysiological differences, limited access to medical care, especially in migrants, may be another contributing factor.

The increased one-year risk of all-cause death and MACE we found in patients with hypertensive emergencies compared to those with hypertensive urgencies supports the findings from previous studies. Although the introduction of more effective antihypertensive treatments has significantly improved the median survival in patients with hypertensive emergencies, that has passed from 39 months during the 1970 to around 60 months in more than 90% of patients by the end of 2016 [13], there is still a significant risk of adverse events in these patients.

One case-control study on 120 normotensive patients, 120 hypertensive patients, and 120 patients with history of hypertensive emergencies form the Netherlands reported that the relative risk (RR) for mortality in those with hypertensive emergencies was higher compared to both normotensive (RR 17.8, 95% CI 2.4–133.6) and hypertensive patients (RR 5.8, 95% CI 1.7–19.8) [6]. A study on 670 individuals presenting for acute severe hypertension in 2015 in France, showed that patients with hypertensive emergencies have a higher one-year mortality compared to those with hypertensive urgencies (38.9% vs. 8.9%, *P* < 0.001) [14]. Finally, an Italian study on 353 consecutive patients presenting with hypertensive crises reported that, after one-year follow-up, compared to patients with hypertensive urgencies, those with hypertensive emergencies had an increased risk of cardiovascular events (HR 3.43, 95% CI 1.7–6.9) and cardiovascular death (HR 13.2, 95% CI 1.57–110.8) [7].

To date, it is unclear why some patients with hypertensive crises develop target organ damage while others do not. The BP values that the vascular system can tolerate vary widely among individuals, depending on the compliance of both resistance and capacitance vessels [12]. However, what it is well established is that the development of acute target organ damages during hypertensive crises represent a strong risk factor for poor prognosis. Indeed, we found that patients with hypertensive crises had a 27% higher risk of all-cause death and a 4-fold increased risk of MACE during the one-year period after the discharge from hospital, compared to those with hypertensive urgencies.

While it was expected that patients who develop intracranial haemorrhage, MI, aorta dissection or rupture, cardiac arrest, acute HF, ischemic stroke, and acute renal failure, would have worse outcomes compared to those without these complications, it is noteworthy that the risk of adverse events was similar across the different types of target organ damage. Sensitivity analysis, which assessed the risk of primary outcomes based on the presence of different prevalent type of target organ damage, revealed that the risk of all-cause death and MACE was comparable among those with the involvement of central nervous system, cardiovascular systems, kidneys, as well as those with multiple complications. This observation has important clinical implications. On the one hand, it highlights the need for more effective preventive strategies in hypertensive patients to avoid the onset of hypertensive crises and to identify those at higher risk. On the other hand, it underscores the importance of effectively managing BP increases in patients presenting with hypertensive crises and establishing appropriate follow-up strategies for those discharged alive from hospital [8].

When analysing the differences in the risk of MACE among various subgroups of patients with hypertensive emergencies compared to those with urgencies, we found that it was higher in patients without previous cardiovascular events and in those without CKD or AF and in those of White ethnicity, compared to those with previous cardiovascular events. CKD, AF or those of Black African ethnicity, respectively. This suggests that the detrimental impact of target organ damage —such as that seen in hypertensive emergencies— may be more pronounced with a greater absolute difference in risk amongst patients with a lower preexisting cardiovascular burden. Indeed, the relative risk observed in our study reflects the effect of hypertensive emergencies against an individual's baseline risk.

Hence, in lower-risk populations – those without prior cardiovascular events, CKD or AF – the occurrence of hypertensive emergencies may result in a proportionally greater increase in cardiovascular outcomes. In contrast, in higher-risk patients, such as those with established cardiovascular disease or CKD, the already elevated baseline risk may partially mask the relative impact of hypertensive emergencies.

A similar principle applies to AF, which exhibits a complex relationship with adverse outcomes in this population. While AF is associated with a markedly increased risk of all-cause death, its impact on MACE appears comparatively attenuated. This divergence may reflect the multifactorial risk conferred by AF, including increased susceptibility to competing noncardiovascular causes of death, potential mitigating effects of anticoagulation on thromboembolic events, and other comorbidities influencing observed outcomes [15].

Furthermore, although Black or African ethnicity was more prevalent among patients with hypertensive emergencies and independently associated with a higher likelihood of progression from hypertensive urgencies to emergencies, White ethnicity was associated with the highest long-term risk of MACE in the context of hypertensive emergencies compared to urgencies. This finding reinforces the notion that the impact of hypertensive emergencies may be more evident in populations with a generally lower baseline absolute cardiovascular risk.

Beyond the increased risk of all-cause death and MACE, patients with hypertensive emergencies also had an increased risk of incident AF compared to those with hypertensive urgencies. Chronic hypertension though hemodynamic changes, neuroendocrine factors, and cardiac remodelling create a favourable environment where AF can onset and perpetuate [16]. Thus, the high prevalence of preexisting hypertension in patients with hypertensive emergencies, may help to explain the high risk of incident AF found in these patients. However, it cannot be excluded that an acute and severe BP increase may have similar effects, especially when additional well known risk factors for AF, such as the target organ damages, are present. Regardless of the origin, both incident and preexisting AF may contribute to the high risk of adverse events in patients with hypertensive emergencies. Furthermore, the risk of MACE in patients with hypertensive emergencies could be even higher than reported in our study. The subgroup analysis suggests a competitive risk between the two primary outcomes in patients with preexisting AF, potentially leading to an underestimated risk of MACE due to their high risk of all-cause death.

Additionally, we found that the incidence of new dialysis treatments over the one-year period after hospital discharge was higher in patients with hypertensive emergencies than in those with hypertensive urgencies. This may represent another key factor contributing to the increased risk of cardiovascular events, as dialysis dependence not only raises the risk of AF but also predisposes patients to other cardiovascular events and complicates the clinical management of preexisting cardiovascular conditions [17,18].

Lastly, we observed that 4% of patients with hypertensive urgencies progressed to hypertensive emergencies within one-year after hospital discharge. No previous studies have specifically investigated this aspect. Thus, these incidence data and the associated clinical phenotypes – characterised by female sex, young age, Black or Asian ethnicity together with a high cardiovascular burden – needs to be confirmed in dedicated prospective studies.

In light of these results, future studies on the clinical course of patients with hypertensive crises should place greater emphasis on the impact of both incident and preexisting AF, as well as dialysis, on the risk of adverse events. There remains a significant gap in knowledge regarding the optimal screening and therapeutic strategies for detecting and managing AF in this setting. Moreover, further research is needed to identify the key risk factors for the progression from hypertensive urgency to hypertensive emergency, allowing for the characterization of clinical phenotypes with varying levels of risk.

### Limitations

Several limitations should be considered. First, the retrospective nature of this study is associated with the possible presence of unmeasured and selection bias. Second, the identification of patients with hypertensive crises, including hypertensive emergencies and urgencies, was based on ICD-10-CM codes. However, these codes may not capture all affected patients and do not allow us to determine the temporal relationship between the hypertensive crisis and related cardiovascular complications (i.e., whether they occurred before or after the crisis). Third, no analysis was conducted on the type of antihypertensive treatment used both before and during hospital admission. Fourth, adverse events occurring outside this healthcare network may not have been recorded, potentially affecting the risk estimates. Fifth, the absence of a control group with well controlled hypertension limits the generalizability of our results to the broader hypertensive population. Sixth, no analysis was stratified by the presence of social disparities. Seventh, when assessing the risk factors for hypertensive emergency progression, we included only demographic data and major cardiovascular risk factors. This provides only a partial overview of the factors involved, as prior cardiovascular events and medical treatments should also be considered in future studies aimed at establishing a comprehensive risk profile. Eighth, the limited follow-up period does not allow us to estimate the risk of adverse events beyond one year, as extending the follow-up would result in a substantial reduction in the number of patients included from the baseline assessment and a significant decrease in statistical power. Lastly, in this study, we used the definition of hypertensive crises – encompassing both hypertensive urgencies and emergencies – based on the ICD-10-CM classification. However, this definition has been recently abandoned by the latest guidelines from the European Society of Cardiology, which now refer to each condition separately rather than as part of the same clinical entity [19].

## CONCLUSION

Patients with hypertensive emergency have a high long-term risk of all-cause death, MACE and incident AF. Preventing the onset of target organ damage in patients with hypertensive crisis is crucial to mitigate their long-term risk of adverse events. This poor prognosis was similar regardless of the type of target organ damage and was independent of age, sex, ethnicity, and preexisting CKD or AF.

## ACKNOWLEDGEMENTS

None.

Contributors: T.B.: conceptualisation, formal analysis, interpretation of results, writing original draft; S.H.M., A.A.A., D.G.B., E.S., A.S. critically revised the manuscript; G.Y.H.L.: supervision, validation, and writing original draft. All authors approved the final version of the manuscript.

Data sharing statement: The data utilized for this study are available on the TriNetX online platform. Access to TriNetX's data requires submitting requests to TriNetX and a data-sharing agreement.

Funding: None.

### Conflicts of interest

G.Y.H.L. is a consultant and speaker for BMS/Pfizer, Boehringer Ingelheim, Daiichi-Sankyo, Anthos. No fees are received personally. He is a National Institute for Health and Care Research (NIHR) Senior Investigator and co-PI of the AFFIRMO project on multimorbidity in AF (grant agreement No 899871), TARGET project on digital twins for personalised management of AF and stroke (grant agreement No. 101136244) and ARISTOTELES project on artificial intelligence for management of chronic long term conditions (grant agreement No. 101080189), which are all funded by the EU's Horizon Europe Research & Innovation programme. All other authors report no disclosures.

## Supplementary Material

Supplemental Digital Content
