# Supplementary material for: Long-term risk of adverse events in patients discharged alive after hospitalization for hypertensive crisis
Source: J Hypertens. 2025 Aug 6;43(10):1703–10. doi: 10.1097/HJH.0000000000004113 (PMC12404630; doi:10.1097/HJH.0000000000004113)

**Long-Term Risk of Adverse Events in Patients Discharged Alive After Hospitalization for Hypertensive Crisis**

Tommaso Bucci, Steven H.M. Lam, Antonios A. Argyris, D. Gareth Beevers,

Eduard Shantsila, Alena Shantsila, Gregory Y. H. Lip

Supplementary material

TriNetX Database

The TriNetX data are sourced from member healthcare organizations (HCOs), originating from their primary electronic health records (EHR) systems. Typically, an HCO is a large academic health center, with data contributions from most of its affiliated facilities. A single HCO often includes multiple facilities, such as main and satellite hospitals. Data are stored on the TriNetX database either via a physical server at the institution’s data center or through a virtual hosted appliance. The TriNetX platform consists of a network of these appliances connected in a federated system. This system allows queries to be broadcast to each appliance, with the results then collected and aggregated. Once data are sent to the network, they are mapped to a standardized set of clinical terminologies and undergo a quality assessment process, including ‘data cleaning’ to exclude records that do not meet TriNetX’s quality standards. The TriNetX database performs an internal and thorough data quality assessment with each data refresh, focusing on conformance, completeness, and plausibility (http://doi.org/10.13063/2327-9214.1244). To ensure HIPAA (Health Insurance Portability and Accountability Act) compliance, clinical patient data are de-identified. The network includes data types such as demographics, diagnoses (coded with ICD-10-CM), procedures (coded in ICD-10-PCS or CPT), and measurements (coded to LOINC). While detailed information on patients’ diagnoses and procedures is available, other variables (such as socioeconomic and lifetime factors) are not comprehensively represented. The advantage of EHR data over insurance claim data is that it includes both insured and uninsured patients. Compared to survey data, EHR data more accurately reflect diagnostic rates within the population that accesses healthcare services, providing a clearer picture of the burden of specific diagnoses on the healthcare system. A primary limitation of relying on diagnoses is that undiagnosed patients who have not sought medical care are not accounted for. Another general limitation of EHR data is that a patient might receive care at different HCOs, and if one HCO is not part of the federated network, portions of their medical records may be missing. Using a network of healthcare organizations helps reduce this issue, but it does not fully eliminate it.

Propensity Score Matched Analyses

Using logistic regression [Logistic Regression from the scikit-learn package in Python (version 3.7)], TriNetX performs a 1:1 greedy nearest neighbor matching model, with a caliper of 0.1 pooled standard deviations. To reduce bias from nearest neighbor algorithms, the order of rows was randomized. Any baseline characteristic with a standardized mean difference between cohorts below 0.1 was considered well-matched (<https://www.tandfonline.com/doi/full/10.1080/00273171.2011.568786>).

Supplementary Table 1. ICD-10-CM codes for inclusion and exclusion criteria in patients with hypertensive emergencies and urgencies.

| Study period: 01^st^ January 2000 – 31^st^ December 2022 | |
| --- | --- |
| **Patients with hypertensive emergencies** | |
| Inclusion criteria | Patients aged ≥ 18 years with the following diagnosis within 30 days before the hospital discharge:   1. Hypertensive emergencies (ICD-10-CM I16.1)   and   1. Central nervous system involvement: ischemic stroke (I63), hypertensive encephalopathy (I67.4), intracerebral hemorrhages (I60 and I61). 2. Cardiovascular system involvement: myocardial infarction (I21), acute heart failure (I50.21, I50.23, I50.31, I50.33, I50.41, I50.43), aortic dissection or rupture (I71.1, I71.3, I71.5, I71.8).   and/or   1. Renal involvement: acute kidney failure (N17) |
| Exclusion criteria | Death before the hospital discharge |
| **Patients with hypertensive urgencies** | |
| Inclusion criteria | Patients aged ≥ 18 years with the following diagnosis within 30 days before the hospital discharge:   1. Hypertensive urgencies (I16.0) |
| Exclusion criteria | Patients aged ≥ 18 years with the following diagnosis within 30 days before the hospital discharge:   1. Central nervous system involvement: ischemic stroke (I63), hypertensive encephalopathy (I67.4), intracerebral hemorrhages (I60 and I61).   and / or   1. Cardiovascular system involvement: myocardial infarction (I21), acute heart failure (I50.21, I50.23, I50.31, I50.33, I50.41, I50.43), aortic dissection or rupture (I71.1, I71.3, I71.5, I71.8).   and / or   1. Death before the hospital discharge |

**Supplementary Table 2.** ICD-10-CM codes for the 1-year risk of all-cause death, thrombotic events, and bleeding.

|  | Diagnosis and ICD-10-CM code |
| --- | --- |
| All-cause death | - Deceased (TriNetX variable) |
| Major Adverse Cardiovascular Events (MACE) | - Myocardial infarction (I21)   and/or   - Acute heart failure (I50.21, I 50.23, I 50.31, I 50.33, I 50.43)   and/or   - Cardiac arrest (I46)   and/or   - Ischemic stroke (I63)   and/or   - Dissection or rupture of aorta (I71.0, I71.1, I71.3, I71.5, and I71.8) |
| Atrial Fibrillation* | - Atrial Fibrillation or flutter (I48) |

*Only for sensitivity analysis.

**Supplementary Table 3**. Risk of primary outcomes according to the type of target organ damage in patients with hypertensive emergencies compared to those with hypertensive urgencies.

|  | All-cause death | | | MACE | | |
| --- | --- | --- | --- | --- | --- | --- |
| (n= number of patients in each group after PSM) | Hypertensive emergencies  N of events (%) | Hypertensive urgencies  N of events (%) | HR (95%CI) | Hypertensive emergencies  N of events (%) | Hypertensive urgencies  N of events (%) | HR (95%CI) |
| At least kidney involvement (n=6,761) | 800 (11.8) | 545 (8.1) | 1.60 (1.44-1.79) | 2,577 (38.1) | 1,012 (15.0) | 3.43 (3.19-3.69) |
| At least CV involvement (n=4,866) | 658 (13.5) | 479 (9.8) | 1.48 (1.31-1.66) | 2,052 (42.2) | 743 (15.3) | 3.75 (3.45-4.08) |
| At least CNS involvement (n=4,077) | 541 (13.3) | 380 (9.3) | 1.58 (1.39-1.80) | 1,626 (39.9) | 576 (14.1) | 3.93 (3.57-4.32) |
| All 3 organs involved (n=975) | 156 (16.0) | 96 (9.8) | 1.83 (1.42-3.36) | 464 (47.6) | 168 (17.2) | 4.25 (3.56-5.07) |

Legend: CI: Confidence interval, CNS: Central Nervous System, CV: Cardiovascular, HR: Hazard Ratio, MACE: Major Adverse Cardiovascular Events.

**Supplementary Table 4.** Subgroup analyses of the risk of all-cause death and major adverse cardiovascular events in patients with hypertensive emergencies versus hypertensive urgencies across clinically relevant groups.

|  | All-cause death | | |  | MACE | | |  |
| --- | --- | --- | --- | --- | --- | --- | --- | --- |
| (n= number of patients in each group after PSM) | Hypertensive emergencies  N of events (%) | Hypertensive urgencies  N of events (%) | HR (95%CI) | P for interaction | Hypertensive emergencies  N of events (%) | Hypertensive urgencies  N of events (%) | HR (95%CI) | P for interaction |
| Males  (n=7,709) | 577 (7.8) | 505 (6.6) | 1.17  (1.04-1.32) | <0.001 | 2,561 (33.2) | 774 (10.0) | 4.1  (3.78-4.44) | 0.725 |
| Females (n=8,912) | 918 (10.3) | 627 (7.0) | 1.55  (1.40-1.71) |  | 2,901 (32.6) | 912 (10.2) | 4.02  (3.73-4.33) |  |
| ≥ 75 years (n=5867) | 923 (15.7) | 677 (11.5) | 1.48  (1.34-1.64) | <0.001 | 1,861 (31.7) | 619 (10.6) | 3.87  (3.53-4.24) | 0.103 |
| < 75 years (n=9,517) | 542 (5.7) | 496 (5.2) | 1.11  (0.99-1.26) |  | 3,104 (32.6) | 892 (9.4) | 4.27  (3.96-4.60) |  |
| CKD  (n=5,223) | 730 (14.0) | 600 (11.5) | 1.27  (1.14-1.41) | 0.198 | 1,860 (35.6) | 932 (17.8) | 2.45  (2.26-2.65) | <0.001 |
| No CKD  (n=11,547) | 786 (6.8) | 590 (5.1) | 1.40  (1.26-1.55) |  | 3,589 (31.1) | 815 (7.1) | 5.56  (5.00-5.88) |  |
| AF  (n=2,905) | 453 (15.6) | 306 (10.5) | 1.62  (1.40-1.87) | 0.005 | 1,060 (36.5) | 449 (15.5) | 3.14  (2.81-3.51) | <0.001 |
| No AF (n=10,081) | 762 (7.6) | 636 (6.3) | 1.25  (1.12-1.39) |  | 3,143 (31.4) | 861 (8.6) | 4.55  (4.21-4.90) |  |
| Whites (n=8,852) | 918 (10.4) | 711 (8.0) | 1.36  (1.23-1.50) | 0.164* | 2,927 (33.1) | 820 (9.3) | 4.54  (4.20-4.91) | <0.001* |
| Black or African Americans (n=4,885) | 322 (6.6) | 276 (5.6) | 1.19  (1.01-1.39) |  | 1,606 (32.9) | 583 (11.9) | 3.35  (3.05-3.68) |  |
| Asians  (n=937) | 70 (7.5) | 43 (4.6) | 1.71  (1.17-2.50) | 0.253* | 298 (31.8) | 97 (10.4) | 3.84  (30.5-4.83) | 0.176* |

Legend: AF: Atrial Fibrillation, CI: Confidence Interval, CKD: Chronic Kidney Disease, HR: Hazard Ratio, MACE: Major Adverse Cardiovascular Events.

*Reference: White ethnicity

**Supplementary Figure 1**. Multivariable Cox regression analysis for risk factors of progression from hypertensive urgency to hypertensive emergency.


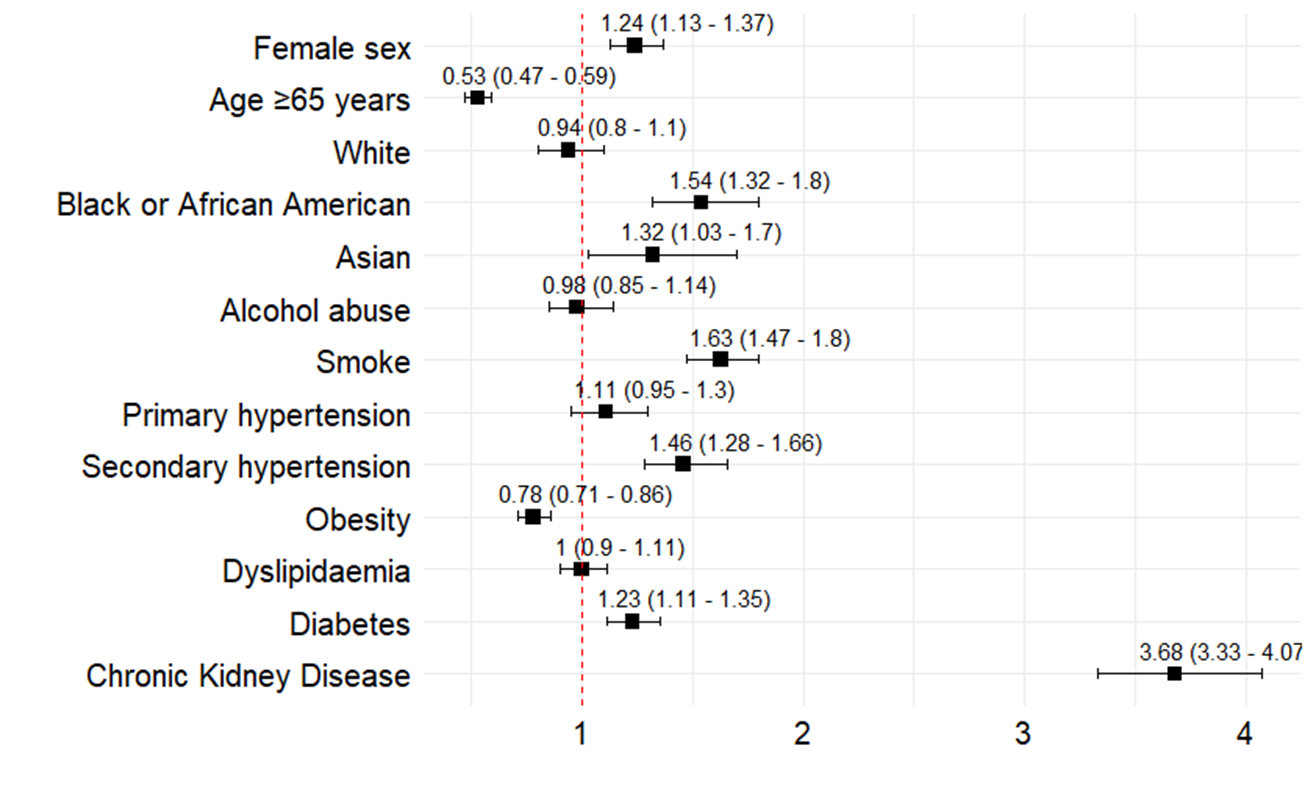

Supplement: Supplemental Digital Content [file jhype-43-1703-s001.docx]
